# TraV: A Genome Context Sensitive Transcriptome Browser

**DOI:** 10.1371/journal.pone.0093677

**Published:** 2014-04-07

**Authors:** Sascha Dietrich, Sandra Wiegand, Heiko Liesegang

**Affiliations:** Abteilung für Angewandte und Genomische Mikrobiologie, Institut für Mikrobiologie und Genetik, Norddeutsches Zentrum für Mikrobielle Genomforschung, Georg-August-Universität Göttingen, Göttingen, Germany; CSIR Institute of Genomics and Integrative Biology, India

## Abstract

Next-generation sequencing (NGS) technologies like Illumina and ABI Solid enable the investigation of transcriptional activities of genomes. While read mapping tools have been continually improved to enable the processing of the increasing number of reads generated by NGS technologies, analysis and visualization tools are struggling with the amount of data they are presented with. Current tools are capable of handling at most two to three datasets simultaneously before they are limited by available memory or due to processing overhead. In order to process fifteen transcriptome sequencing experiments of *Bacillus licheniformis* DSM13 obtained in a previous study, we developed TraV, a RNA-Seq analysis and visualization tool. The analytical methods are designed for prokaryotic RNA-seq experiments. TraV calculates single nucleotide activities from the mapping information to visualize and analyze multiple transcriptome sequencing experiments. The use of nucleotide activities instead of single read mapping information is highly memory efficient without incurring a processing overhead. TraV is available at http://appmibio.uni-goettingen.de/index.php?sec=serv.

## Introduction

The possibility to sequence complete transcriptomes (RNA-Seq) opens a new level of quality of transcriptomics [1]. The development of next generation sequencing (NGS) methods [2] enabled the quantitative strand-specific investigation of transcriptional activities of genomes at single nucleotide resolution [3], [4]. The discovery of new regulatory RNA features like small RNAs, riboswitches and antisense transcripts revealed the existence of an RNA based regulation layer in prokaryotic genomes [5], [6]. A detailed analysis of highly resolved data on transcriptionally active genome loci should therefore enable the identification of these regulators on a whole genome scale. A comprehensive workflow in a microbial transcriptome experiment may comprise four steps: (i) production of sequence data from an RNA sample, (ii) filtering of bad quality reads and reads for rRNA and tRNA sequences, (iii) strand-specific mapping of the remaining RNA-derived sequences to the genome, and finally (iv) functional analysis of the transcriptionally active genomic regions in their physiological context. A deep sequencing experiment on a microbial genome with Illumina technology may result in several million reads per experiment [7]–[9]. This sheer amount of data is challenging with regards to read mapping as well as detailed analysis. Obviously, sophisticated bioinformatics tools are crucial to enable convenient and rapid RNA-Seq data analysis. Whereas NGS techniques like 454, Illumina and ABI Solid have continuously advanced over the years, approaches to improve or develop bioinformatics tools to handle the generated data have focused almost exclusively on mapping of the sequences to a genomic backbone, e.g. SSAHA2 [10], bowtie2 [11] and/or BWA [12]. Current visualization tools like Artemis [13], SAMSCOPE [14] or Integrative Genomics Viewer [15] focus on single or few parallel datasets and face performance issues due to the handling of single read mapping information. Therefore, the analysis of multiple datasets in parallel remains difficult, thus demanding further developments in the area of visualization and automated analysis. Here, we introduce TraV (**Tra**nscriptome **V**iewer), a freely available tool which provides support in organization and analysis of multiple transcriptome datasets in relation to the corresponding genomic context. TraV focuses on the identification of regions of transcriptional activity in correspondence to known genes like 5′ and 3′ untranslated regions (UTRs), transcripts that do not correspond to known genomic features, antisense transcripts and transcription start sites (TSS). TraV's ability to process many RNA-Seq data sets simultaneously enables the comparison of many different experimental conditions at the same time. The handling of multiple RNA-Seq datasets is based upon a data abstraction which transforms read mapping data into single base transcriptional activities of the genome. In case single read mapping information is required other tools have to be applied. Thereby, the tool facilitates the search for novel features based on comparative RNA-Seq analysis. TraV's capabilities make the tool an appropriate choice for the comparative analysis of multiple transcriptome experiments with focus on the transcriptional activities of corresponding genome loci from different experiments.

## Materials and Methods

### Calculation of base activity counts

TraV uses single base resolution coverage counts for both positive and negative strand as basis for all calculations and graphical presentations of mapping information, a method firstly described by Wurtzel *et al.* [4]. The coverage counts are calculated from a SAM mapping file [16], obtainable from currently available single read mappers like e.g. bowtie2 [11]. The SAMtoTDS tool provided with TraV can be used to convert SAM files into TraV's innate TDS format (see below for details). For each successfully mapped read, the associated base coverage counts for regions covered by the read are increased by one. To constitute a successful mapping, a read has to be uniquely mapped, meaning that it has only one optimal mapping position in the genome. In case of multiple best mapping locations, a read is considered multi-mapped and will not be included in the base coverage calculation. After all mapped reads have been processed; the resulting base coverage strings serve as an abstracted representation of the mapping. This procedure greatly reduces the memory required to handle such transcriptome mapping data and thereby allows TraV to deal with multiple datasets simultaneously.

### Data analysis

The analytical methods embodied in TraV are designed for the identification of regions of transcriptional activity fulfilling a set of constraints. These regions of transcriptional activity can be indicative of novel RNA features. The methods are implemented as background tools and do not interfere with the functionality of the display view. Each analytical method can be performed independently on loaded data sets. Results are provided either as tab-separated value files or General Feature Format (GFF version 3) formatted files. TraV's analytical methods work on a single nucleotide resolution by avoiding sliding window-based approaches. This allows TraV to make predictions accurate to a single base, giving the maximal possible precision of the analytical approaches like e.g. transcription start site (TSS) predictions. The comparison of RNA-Seq experiments from different conditions requires normalization of the mapped data. To achieve this goal, Mortazavi *et al.* [17] introduced the read oriented RPKM values. RPKMs represent the number of reads mapped for any transcriptionally active region normalized against the total number of reads per experimental condition. As TraV does not use single read information, RPKMs cannot be calculated. TraV instead uses nucleotide activities per kilobase of exon model per million mapped reads (NPKM) values (Equation 1 and [9]) to represent transcriptional activity of all identified regions of transcriptional activity in its analytical methods. 
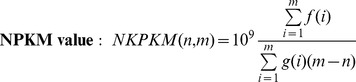
(1) Where *n* and *m* are the start and stop of the region of interest, *f(i)* is the base activity of base *i* on a specific strand and *g(i)* is the sum of the activities of base *i* of positive and negative strands [9].

### Implementation

TraV is implemented as a JAVA web application for Linux-based webservers capable of providing a java container. Runtime-critical and memory-limited procedures have been implemented in C++. For data storage and retrieval a PostgreSQL database (PostgreSQL version 8.4 or higher) is used. A dedicated user management has been established which consists of two administrative and one application level. The user management is implemented via a WWW interface with dedicated user accounts. TraV features three different levels of access: (i) the admin user who may create and delete user accounts and has the ability to create new projects, (ii) administrative users who may import, export and delete transcriptome data sets for their assigned projects and (iii) standard users who may view and analyze the transcriptome data sets of their projects. The webserver-based implementation with differing levels of access makes TraV a good solution for cooperative projects. TraV centralizes data storage and processing for workgroups and provides access to the server-based environment for workstations that do not provide the hardware to cope with the amount of data generated in whole genome transcriptome sequencing experiments. The secure user password restricted WWW access was designed to enable users to work from any reliable internet connection. TraV may use annotated genome information, either as EMBL- or as GenBank-formatted files, to generate the genomic context of transcriptionally active regions. It is possible to assign multiple EMBL or GenBank-formatted files to a single genome project, thus enabling projects that include strains with multiple replicons or draft genomes.

#### Graphical user interface

**Figure 1:**
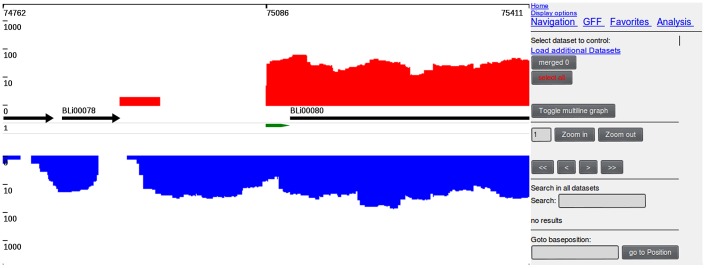
Main display of TraV. Transcriptional activity is depicted by graphs (red graph representing positive strand, blue representing negative strand). Annotated genome information is represented by black arrows. User-provided GFF-based annotations are represented in tracks between the original genome information by coloured arrows (green arrow in this figure).

**Figure 2:**
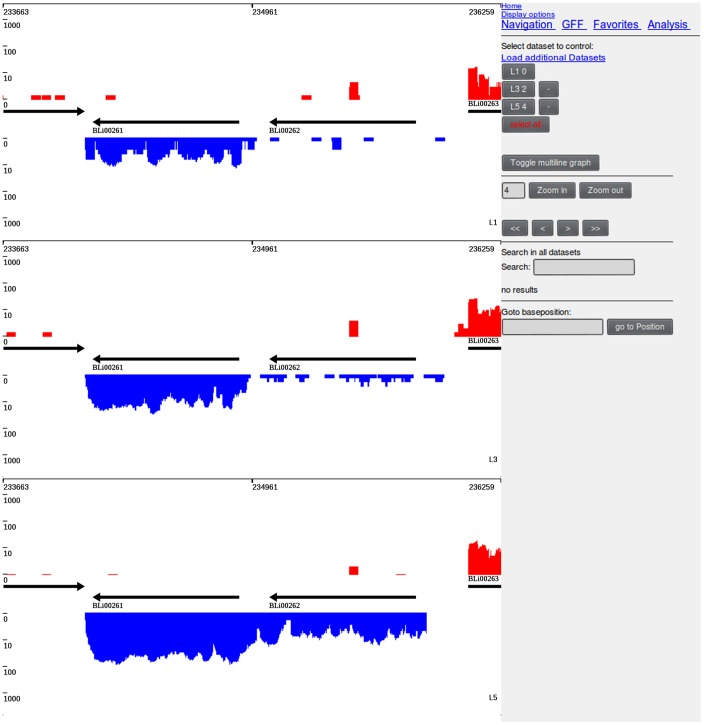
Differential expression. Differential expression of genes BLi00261 and BLi00262 at selected time points of a fermentative process. Dataset L-I shows the transcriptional activity of *B. licheniformis* DSM13 at the early exponential growth phase, L-III shows the transcriptional activity at the end of the exponential growth phase and L-V shows the transcriptional activity at the late stationary phase.

**Figure 3:**
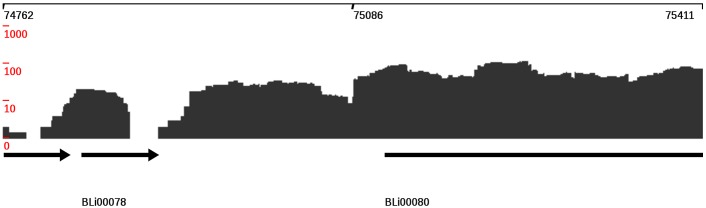
Strand unspecific mode of TraV. In case that the available mapped read data does not contain strand specificity information TraV can be switched into the strand unspecific mode in which all mapped reads are summed up to an general activity of a genome locus.

**Figure 4:**
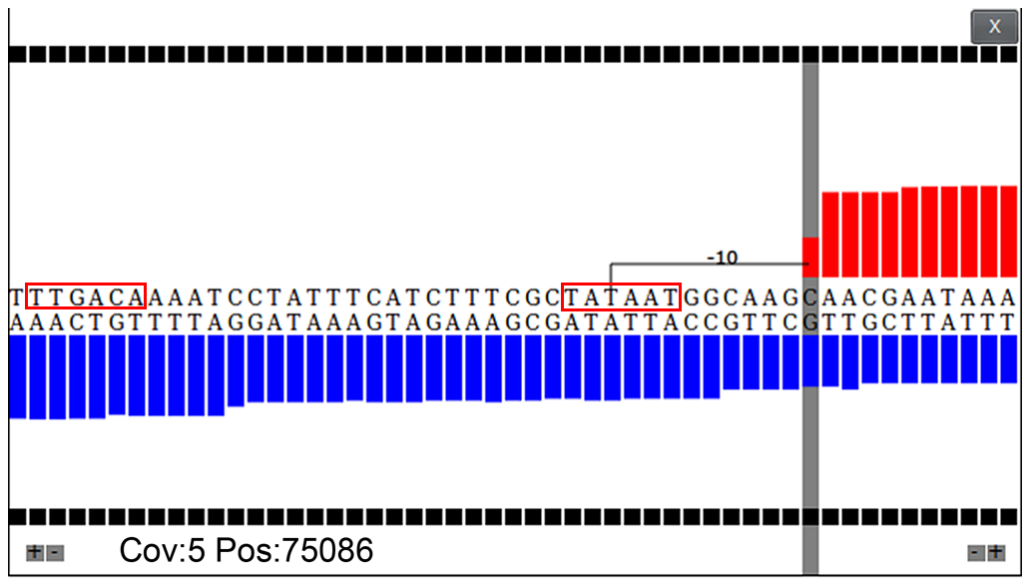
Magnification view of TraV. Transcriptional activities are displayed at single nucleotide resolution. The interactive graphic allows access to coverage information as well as to distinct base distances between the selected base (grey marker) and any other base of the displayed sequence. In the example perfect −10 and −35 boxes of a sigma70 promoter are visible.

The display view represents the main working interface of TraV (Figure 1). The interface allows navigating, zooming, searching by specific genomic features, loading of additional transcriptome data sets for comparison (Figure 2) as well as accessing the analytical methods. All displayed plots are log scaled to enable the view of a wide range of possible transcriptional activities within the display. Strand-specificity is addressed by two different coverage plots: red and blue mark the transcriptional activity of the positive and negative strand, respectively, whereby positive and negative represent the orientation of the genome in relation to *dnaA*. TraV has been developed for RNA-Seq protocols which generate strand-specific data, thus the TraV default working mode is “strand-specific”. However, data sets may not always contain strand-specific information. To enable the analysis of strand-unspecific data, a corresponding work mode has been implemented (Figure 3). Within the strand-unspecific mode, coverage counts from both strands are summed up. To gain the full value of the single nucleotide resolution of RNA-Seq data, TraV contains a magnification view, which displays sequence information in context to their transcriptional activity (Figure 4). For instance, this function proved suitable for manual promoter pattern searches guided by TSS. The graphical viewer of the transcriptome and the genomic features are interactive and display information on coverage and genome position upon mouse input events. All graphics comply with the Scalable Vector Graphics (SVG) standard. Should rasterized graphics be required, TraV can convert the SVG graphics to Portable Network Graphics (PNG) images. TraV supports user-provided annotations in the form of GFF3-formatted files. Each loaded annotation set is displayed by coloured arrows between the provided genome annotations. GFF-encoded loci may be used for feature-oriented navigation as well as input for annotation-dependent analytical methods. To enable comparisons of different experiments with different sequencing efficiencies within the interface, TraV contains a normalization method for data sets depending on their mapped read count. The normalization factor x is calculated specifically for each loaded dataset (Equation 2: Normalization factor: 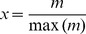). The factor x exclusively scales the graphical representation of activity plots to the data set with the highest read number. Currently, TraV does not offer a normalization method to account for sequencer bias such as Illumina read biases deriving from random hexamer priming, as described by Risso *et al.* [18] and Hansen *et al.* [19].
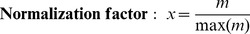
(2)Where x is the normalization factor for the current dataset applied to each base activity, m is the number of mapped reads in the current dataset and max(m) is the greatest amount of mapped reads of all currently loaded data sets.

#### Analytical methods

TraV has been designed for the identification of regions of transcriptional activity such as 5′ and 3′ untranslated regions [20], transcriptional activities of non-annotated genome loci (which may encode sRNAs or hitherto unknown protein genes) and antisense transcripts. Furthermore, transcription pattern search for sharp increases in transcription activity can be used to identify transcription start sites. To represent transcriptional activities, TraV can calculate NPKM values for all identified regions as well as already annotated genomic or user-provided features.

**Figure 5:**
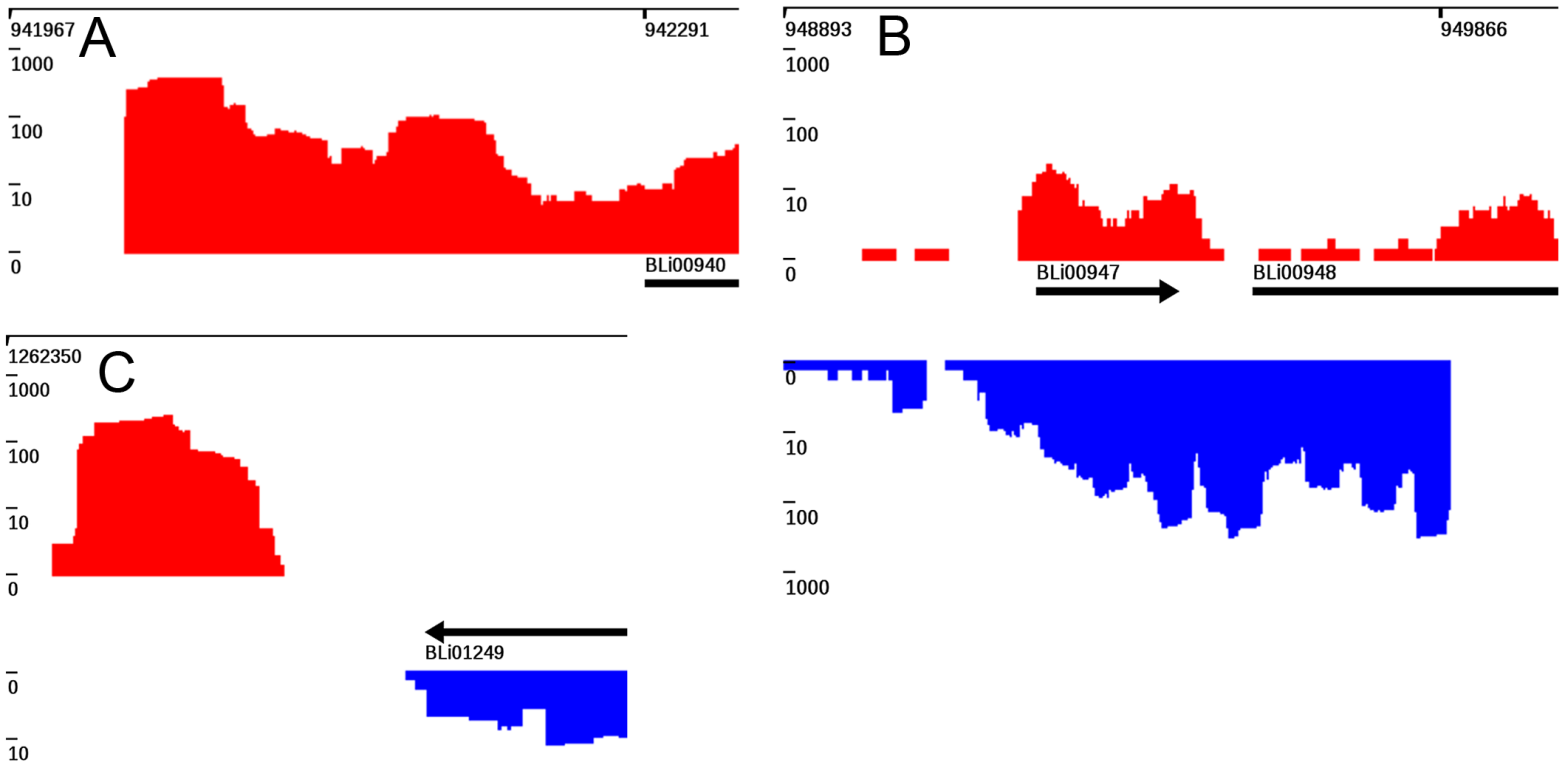
Patterns visualized by TraV. (**A**) 5′UTR of annotated gene feature. (**B**) Antisense transcript. (**C**) Transcriptional activity at a non-annotated region (free transcripts). A, B and C also show strong increases in transcriptional activity, which TraV predicts as transcription start sites. All graphs shown represent data from RNA-Seq experiments performed on *B. licheniformis* DSM13 [9] (indicated by red boxes). All graphs shown represent data from RNA-Seq experiments performed on *B. licheniformis* DSM13 [9].

TraV offers five analytical methods to accomplish the detection of RNA (Figure 5). Since some analytic procedures can consume a considerable amount of computing power, it is possible to choose which data sets should be included for further analysis. In case multiple RNA-Seq data sets are selected, one merged dataset accumulating all available data is generated from these data sets. TraV adds the base transcriptional activities of each selected dataset for each base position to create the merged dataset. This approach allows replicates to close gaps in the sequencing coverage and therefore improves the accuracy of the predictions. Merging data sets originating from different growth phases or environmental conditions enables a combined search for overlapping as well as for differentially expressed features. However, this approach comes with the potential drawback that features like alternative transcription start sites or alternative termination sites become less obvious. Therefore, in cases where the merging of the data sets might obfuscate interesting features, only single data sets should be used for analysis. Prior to merging different data sets, TraV performs a noise filtering on each dataset by removing singularly mapped reads which never intersect with at least one other mapped read from the dataset. This is accomplished by searching for regions of transcriptional activity going from zero to one and back to zero activity without ever encountering an activity greater than one. TraV does not check for a minimum or maximum length of such a region since the different sequencing methods are producing reads of varying length. Subsequently, TraV performs the described merging process and applies the selected analytical method to the merged dataset. For each region identified by an analytical method, TraV calculates the NPKMs from the original data sets instead of the merged set. This allows a comparison of the transcriptional activity of a region between the different data sets. All analytical methods, with the exception of “Transcription Start Site prediction” can use either the replicon provided annotations or user-provided annotations in form of imported GFFs.

- **Calculate NPKM Values** determines NPKMs for annotated regions, which may also include any GFF-definable features (i.e. genes, operons or phages). NPKMs enable impromptu comparisons of the transcriptional expression strength of features. These comparisons may indicate candidate genes for more sophisticated, statistical methods like e.g. baySeq [21].
- **3′ and 5′UTR Search** identifies regions of uninterrupted transcriptional activity entering or leaving an annotation. The algorithm searches regions of transcriptional activity flanking annotated features. The start and stop of transcriptional activity has to lie outside of an annotated feature on the same strand.
- **Free Transcript Search** scans the transcriptome for regions of transcriptional activity that do not intersect with any annotated genomic features on either positive or negative strand. Therefore this method is suitable to scan for genes which might correspond to not yet annotated sRNAs or protein-coding genes.
- **Antisense Transcript Search** is functionally similar to “Free Transcript Search”. In contrast to the constraints of a free transcript, an antisense transcript requires an annotation on the opposite strand of its genomic location. Since the method requires strand-specific transcriptome data, it is unavailable in TraV's strand-unspecific mode.
- **Transcription Start Site Prediction** predicts candidates for TSS based on the identification of strong increases in transcriptional activity over a very short distance. This is done by calculating the slope of the transcriptional activity graph at any given position of the genome. This method is controlled by two values: the minimal height of the increase in transcriptional activity that constitutes the minimum height to define a positive hit and the slope distance which defines how many bases the slope should include. TraV offers a function to suggest an appropriate slope for a dataset. This function does a 5′UTR search and checks the distance necessary to obtain a positive hit with the current minimum height. The suggested standard value for the minimal height of three proved to be a good compromise between accuracy and noise in our test cases. Obviously, this method is dependent on the coverage of the sequencing experiment and insufficient coverage information may lead to gaps in transcript coverage to be misinterpreted as TSS candidates.

Please note that, depending on operon structure and regulation of transcription units, the different analytical methods may identify regions that belong to another feature class. For instance, the “Free Transcript Search” method may identify features that are actually *cis*-regulatory elements inhibiting downstream transcription which are therefore not connected to the respective gene. Therefore each region identified by the analytical methods has to be considered as a candidate for a RNA feature and the biological relevance has to be evaluated carefully.

#### TDS format and SAMtoTDS converter

TraV employs the generic TDS flat file data format to describe and save transcriptome mappings. The format consists of the following three sections: (i) a count data section which stores the number of mapped and unmapped reads and the total activity count of the RNA-Seq experiment. The mapped reads count is used by TraV for normalization of the graphical representation of different RNA-Seq experiments. The total activity count is used for the calculation of NPKM values. (ii) A coverage section, introduced by the string \\COV. In this section, the mapping is represented by transcriptional activity values per base. Every line in this section represents the coverage data of one base. (iii) An optional \\READS section can be used to provide the single read mapping information. TraV does not use single read mappings, however, as downstream analysis tools like mpileup [16] or GATK [22] require single read mapping information, this data can be included. TraV provides a command line conversion tool for SAM-formatted [16] mappings to generate its TDS import data format. This tool uses the CIGAR and bit flag information of the SAM format to determine successful read mappings. SAMtoTDS can use the CIGAR string information to apply a sequence similarity filter. This function is essential since some mappers do not apply a whole sequence length similarity filter. SAMtoTDS has been successfully used on SAM files generated by bowtie2, BWA and SSAHA2. This conversion step from SAM to TDS is done outside of TraV to conserve bandwidth of the server as the resulting TDS files are much smaller than the original SAM files.

## Results and Discussion

TraV was developed and extensively tested for the analysis of RNA-Seq experiments on *Bacillus licheniformis* DSM13 during an industrial fermentation process [9]. The tested RNA-Seq data was sequenced using an Illumina HiSeq 2000 machine with a read length of 50 nucleotides and is available in the Sequence Read Archive (SRA) under accession no. SRP018744. Mapping was performed using the Wurtzel *et al.* [4] BLAST-based method with a minimum sequence similarity of 98% for the complete read length. TraV was furthermore used to successfully visualize eukaryotic RNA-Seq data from *Schizosaccharomyces pombe* (SRA accession no. PRJEB3065). Please note: the analytical methods are currently only applicable to prokaryotic data since TraV does not yet have methods to deal with splicing events.

### Transcriptionally active region evaluation

Predicted UTR and Free/Antisense transcript candidates were checked with the Rfam [23] covariance models for known structured RNAs. The results of this analysis are presented in Table 1. These predictions were used as foundation for a detailed manual curation and characterization process of regulatory RNA candidates [9]. Among the identified candidates are expected RNA genes like the 6S RNAs, the tmRNA as well as orthologous riboswitches described in the Rfam database. However, beside the elements with an Rfam assignment, a great number of transcribed RNA features without known function with a size greater than 100 nt have been identified which may contain interesting candidates for novel regulatory RNA features.

**Table 1:** Identified UTRs and Free Transcripts in *B. licheniformis* DSM13.

	3′UTR	5′UTR	Free Transcripts	Antisense Transcripts
Identified with TraV	1396	1404	476	3777
Candidates >100 nt	581	446	124	1933
Candidates with Rfam hits	78	27	10	212

### Visualization and Memory Management

Tools like T-ACE [24], Tablet [25] and SAMSCOPE [14] use indexed single reads and display the mapping information based on these single reads. Tools like T-ACE [24] suggest upper limits to their input data due to these limitations (60,000 bp contig size with a suggested maximum of 100,000 mapped reads). This reduces their usability with increasing contig sizes commonly used in whole genome RNA-Seq experiments. With the application of high coverage generating NGS techniques they are inevitably challenged by memory consumption issues. Programs like Tablet, SAMSCOPE or Artemis circumvent this issue by using the indexing information of BAM [16] to stream the loading and unloading of read information based on the displayed genomic region. This approach reduces the memory footprint but causes computational overhead due to the streamed loading mechanism leading to bad responsiveness when dealing with multiple datasets at the same time and to possible memory limitations at big window sizes. In contrast, TraV loads the whole data set and keeps it available in memory; therefore the tool does not incur stream loading congestions. The memory requirements on our test data resulted in approximately 50 Mbyte per data set with TraV when loaded in the working environment. In our tests TraV still works with 15 simultaneously loaded data sets in a comparative analysis on a workstation with 6 Gbyte RAM. Since TraV sacrifices single read information, it is unsuitable for data analysis dependent on information derived from base differences of single reads like single nucleotide polymorphism (SNP) studies, phase variation analysis or methylome studies by bisulfate sequencing.

### Transcription start site prediction

RNA-Seq-based transcription start site prediction has been shown to be efficient by Sharma *et al.* [26], [27]. The method described therein generates region lists within a 500 nt sliding window based on the comparison of transcriptional activities from corresponding RNA-Seq and differential RNA-Seq data sets, which were then manually evaluated. Differential RNA-Seq is a specialized version of RNA-Seq which is designed to identify microbial transcriptions start sites [28]. It is based on a selective digestion of all transcripts which do not represent primary transcripts. In contrast, the TSS prediction by TraV generates TSS candidate lists of single nucleotide resolution on single data sets from high coverage RNA-Seq or preferably differential RNA-Seq data sets, which have to be evaluated by a biological expert. Furthermore, the TraV approach is not dependent on the existence of corresponding RNA-Seq and differential RNA-Seq data sets and can thus be applied to a single deep sequencing experiment. However, TraV's TSS prediction is directly dependent on the coverage; therefore an application of the data of Sharma *et al.* resulted in an accelerated error rate of artefact TSSs due to mapping gaps within transcriptionally active regions. Therefore TraV is not applicable for data sets with an insufficient coverage.

## Conclusion

The hereby introduced RNA-Seq analysis software package TraV includes an import function for mapping data from SAM-formatted mappings, five prediction tools to identify RNA features and a memory efficient transcriptome visualization engine addressing the typical work flow of a comparative RNA-Seq analysis. TraV has been successfully applied to Illumina generated data sets consisting of fifteen RNA-Seq and five differential RNA-Seq records [9]. The analytical tools of TraV identified previously missing protein genes and RNA-based regulatory features like e.g. riboswitches or regulatory RNAs. TraV is inapplicable for single read based data as necessary in SNP analysis, phase variations or bisulfate sequencing. TraV is positioned as a tool focused on comparative high coverage transcriptome mappings on well-polished and annotated microbial genomes. TraV operates simultaneously on multiple RNA-Seq mappings and provides analysis functions that focus on whole contigs rather than limited areas. The program's primary purpose is the global identification of RNA-based regulators within a genome and providing researchers with automatically generated candidate lists and interactive evaluation tools.
